# 

**Published:** 1868-01-15

**Authors:** 


VOL. XXV. NUMBER 2.

### THE CHICAGO MEDICAL JOURNAL.

EDITED BY

J. ADAMS ALLEN, M.D., LL.D.

JANUARY 15, 1868.

CHICAGO:

CHURCH, GOODMAN AND DONNELLEY, STEAM BOOK AND JOB PRINTERS.

108 AND 110 DEARBORN STREET.

